# A study of home deaths in Japan from 1951 to 2002

**DOI:** 10.1186/1472-684X-5-2

**Published:** 2006-03-09

**Authors:** Limin Yang, Naoko Sakamoto, Eiji Marui

**Affiliations:** 1 Department of Public Health, Graduate School of Medicine, Juntendo University, Japan. 2-1-1 Hongo, Bunkyo-ku, Tokyo 113-8421, Japan

## Abstract

**Background:**

Several surveys in Japan have indicated that most terminally ill Japanese patients would prefer to die at home or in a homelike setting. However, there is a great disparity between this stated preference and the reality, since most Japanese die in hospital. We report here national changes in home deaths in Japan over the last 5 decades. Using prefecture data, we also examined the factors in the medical service associated with home death in Japan.

**Methods:**

Published data on place of death was obtained from the vital statistics compiled by the Ministry of Health, Labor and Welfare of Japan. We analyzed trends of home deaths from 1951 to 2002, and describe the changes in the proportion of home deaths by region, sex, age, and cause of death. Joinpoint regression analysis was used for trend analysis. Logistic regression analysis was performed to identify secular trends in home deaths, and the impact of age, sex, year of deaths and cause of deaths on home death. We also examined the association between home death and medical service factors by multiple regression analysis, using home death rate by prefectures in 2002 as a dependent variable.

**Results:**

A significant decrease in the percentage of patients dying at home was observed in the results of joinpoint regression analysis. Older patients and males were more likely to die at home. Patients who died from cancer were less likely to die at home. The results of multiple regression analysis indicated that home death was related to the number of beds in hospital, ratio of daily occupied beds in general hospital, the number of families in which the elderly were living alone, and dwelling rooms.

**Conclusion:**

The pattern of the place of death has not only been determined by social and demographic characteristics of the decedent, but also associated with the medical service in the community.

## Background

During the past 2 decades, end-of-life care has become an increasingly high-profile issue. One of the important issues has been *where *people die. End-of-life care is concerned with meeting patients' and families' wishes of place of death. Several studies [1-4] have found that 50–70% of cancer patients would prefer to be cared for at home and to die there. Despite aggressive efforts to facilitate and encourage death at home in Western countries, only 20–30% of cancer patients die at home [5-10]. Surveys in Japan [11] also indicate that most Japanese would prefer to die at home or in a homelike setting, when they are terminally ill. Similarly, there is a great disparity between the preference to die at home and the fact that most Japanese die in hospital. In 2000, >70% of deaths occurred in hospitals, compared with < 15% of deaths at home.

The issues of place of death have been studied extensively in other countries [4,12-24]. The place of death appears to be determined by multiple factors, such as individual characteristics, disease processes, family and general practitioner's support, social and economic support, and health-care system structure. Individual characteristics associated with place of death include age [5,21], gender, racial, and marital status [22-24]. Factors related to social and economic status include education, family income, deprivation, and social class [5,21]. Regional differences in utilization and availability of health care services and scope of coverage are also related to place of death [12]. However, many of the reported studies were conducted in developed Western countries, and therefore these results may not be applicable to the Japanese population. Previous studies have often been limited to small regions or groups, and hence population-based studies examining home deaths and determinants are lacking in Japan.

In this report, we describe the national changes in home deaths in Japan over 5 decades. Using prefecture data, we also examined some of the main social factors associated with home death in Japan.

## Methods

We obtained published data on place of death from the vital statistics compiled by the Ministry of Health, Labor and Welfare of Japan. We analyzed the trend of home deaths data from 1951 to 2002. Prior to 1988, the categories for place of death used in the vital statistics were as follows: hospital, clinic, maternity home, home, and others. A category of "health service facilities for the elderly" was included in 1989, and "home for the elderly" was added in 1995.

[*N.B. Health service facilities for the elderly means a type of long-term care insurance facility authorized by the Long-term Care Insurance Law, and providing services after admission. Residents receive care in daily life, such as eating, bathing, toileting and other activities, functional training, health management, and medical care are based on the service plan of the facility. Physicians are not stationed there, and medical procedures are rarely performed on site. Therefore, the elderly who require treatment by physicians cannot be admitted. Home for the elderly is the general term for the nursing home for the elderly, special nursing home for the elderly, low-cost home for the elderly, and private nursing home for the elderly authorized by the Welfare Law for the Aged. In this facility, the elderly in the community can receive various types of counseling, support for the promotion of health, cultural improvement, and recreation, and various other services for healthy bright living*.]

For the purpose of this analysis, we only examined the change of home deaths and hospital deaths, because these two categories account for ~90% of total deaths, and the place of death was mostly limited to home and hospital. This report presents total percentage of home death from 1951 to 2002, for age starting in 1980, for course of death starting from 1955, and for region for full time period. We analyzed trends in the proportion of home deaths by region, age, and causes of deaths using Joinpoint regression analysis. This method provides an objective means for evaluating trends in data collected over time. Kim et al. provide a detailed description of statistical theory in term of Joinpoint regression analysis [25]. Trend analysis was conducted by Joinpoint regression program 3.0 (National Cancer Institute, USA) [26]. The software takes trend data and fit the smallest number of change-points supported by the data, that is, where significant shifts of the annual percent change (APC) are observed. The adjacent lines meet at a point called a joinpoint. Each joinpoint denotes a statistically change in the trend. For these analyses a maximum of four joinpoint and five line segments were allowed for each long-term trend. In addition, each segment had to contain at least two observed data points (years), and no segment could begin or end closer than two data points from the beginning or end of the data series. The regression line segments between the joinpoints, are on a logarithmic scale so that the APC for the segment, the slope of the line, can be interpreted. We used the constant variance option for the software.

Second, multivariate logistic regression was performed to identify changes in home deaths, and to examine the impact of age, gender, death year and cause of death on home deaths. For this analysis, we used data from 1980 to 2002, because we could only obtain the summary table data regarding the deaths from leading cause of death by sex, place of occurrence, and age from 1980. The age categorization was divided into 4 groups (< 65, 65–74, 75–84, > 85). Causes of death in the model included the 3 leading causes (cancer, chronic heart disease, and cerebrovascular disease).

Third, using home death rate by prefectures in 2002 as dependent variable, we conducted a multiple regression analysis to evaluate the impact of social and medical service factors on home deaths. We hypothesize the variables involving the utility, availability of health care services and living environments have in relationship to place of death. The following variables were used as independent variables: the number of general hospitals (per 100,000 persons), the number of beds in hospital (per 100,000 persons), ratio of daily occupied beds in general hospital, the number of families in which the elderly were living alone (per 100 households with person 65 years or older), dwelling rooms (per dwelling), health service facilities for the elderly (per 100,000 persons 65 years old and over). Data on these indicators were obtained from the Survey of Medical Institutions, Hospital Report, Population Census and Survey of Institutions and Establishment for long-term care. All indicators used here were reliable, because all these surveys conducted at national level, and by Japanese government agency. The detailed variable resources showed in Table 1.

**Table 1:** Variable resources

**Variables**	**Resource**	**Year**	**Operating institution**
The number of general hospitals (per 100,000 persons)	Survey of Medical Institutions,	2002	Ministry of Health, Labor and welfare
The number of beds in hospital (per 100,000 persons)	Hospital Report	2002	Ministry of Health, Labor and welfare
Ratio of daily occupied beds in general hospital	Hospital Report	2002	Ministry of Health, Labor and welfare
The number of families in which the elderly were living alone (per 100 households with person 65 years or older)	Population Census	2002	Ministry of Public Management, Home Affairs, Posts and Telecommunications
Dwelling rooms (per dwelling)	Population Census	2002	Ministry of Public Management, Home Affairs, Posts and Telecommunications
Health service facilities for the elderly (per 100,000 persons 65 years old and over)	Survey of Institutions and Establishment for long-term care	2002	Ministry of Health, Labor and welfare

Logistic regression analysis and multiple regression analysis were performed by SPSS11.0.

## Results

**Figure 1:**
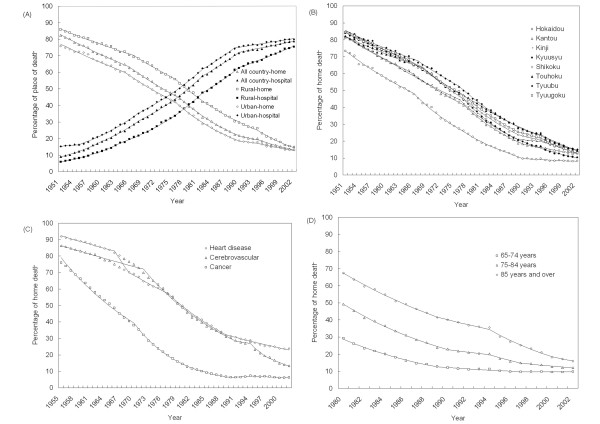
**Trend of deaths at home in Japan. **(A)death at home or hospital by all country, urban and rural from 1951- 2002. (B) by region from 1951–2002. (C) by leading cause of death (cerebrovascular, cancer and heart disease) from 1955–2002. (D) by age (over 65 years for three leading cause of death: cerebrovascular, cancer and heart disease) from 1980–2002. The solid line shows the selected joinpoint model fit for the proportion of place of death.

Trends of home deaths are shown in Table 2 and Figure 1. Figure 1(A) presents the trends of home deaths in all nations and in urban/rural areas. The proportion of home deaths fell gradually from a high of ~82% in 1951 to a low of 13% in 2002. Deaths occurring at hospital correspondingly increased over this period. The selected model of trends in the home death rate has 4 joinpoints (5 line segments). The significant joinpoints showed in 1966, 1976, 1989 and 1994 with significant declining annual percentage changes (APCs) from 1951 to 1966 (-9.0 percent), from 1966 to 1976 (-6.0 percent), from 1976 to 1989 (-5.0 percent), from1989 to 1994 (-4.0 percent) and from 1994 to 2002 (-9.0 percent). Home deaths also declined for both urban and rural locations.

**Table 2:** Statistical significant of trends from joinpoint analysis of percentage of place of death by age, causes of death and regions.

**Characteristics**	**Trend 1**		**Trend 2**		**Trend 3**		**Trend 4**		**Trend 5**	
	**Years**	**APC**^1^	**Years**	**APC**	**Years**	**APC**	**Years**	**APC**	**Years**	**APC**

All country										
Home	1951–1966	-1.77*	1966–1977	-3.17*	1977–1990	-5.46*	1990–1994	-2.78*	1994–2002	-4.80*
Hospital	1951–1960	8.28*	1960–1968	6.64*	1968–1982	4.65*	1982–1990	2.83*	1990–2002	0.76*
Rural-Home	1951–1967	-1.28*	1967–1976	-2.48*	1976–1994	-4.41*	1994–2002	-6.73*		
Rural -Hospital	1951–1964	8.56*	1964–1982	5.77*	1982–1990	3.54*	1990–2002	1.58*		
Urban-Home	1951–1965	-1.76*	1965–1976	-3.19*	1976–1990	-5.76*	1990–1994	-1.89	1994–2002	-3.74*
Urban-Hospital	1951–1955	1.46*	1955–1968	6.11*	1968–1982	4.06*	1982–1989	2.76*	1989–2002	0.56*
Regions										
Hokaido	1951–1966	-2.74*	1966–1977	-5.56*	1977–1987	-6.70*	1987–1990	-8.82*	1990–2002	-1.60*
Kantou	1951–1965	-2.08*	1965–1977	-3.19*	1977–1990	-6.09*	1990–2002	-3.05*		
Kinji	1951–1962	-2.07*	1962–1976	-2.62*	1976–1989	-5.64*	1989–1994	-1.24*	1994–2002	-3.62*
Kyuusyu	1951–1968	-1.65*	1968–1977	-3.74*	1977–1988	-6.24*	1988–2002	-5.46*		
Shikoku	1951–1968	-1.71*	1968–1978	-3.85*	1978–1989	-5.21*	1989–1994	-2.99*	1994–2002	-5.51*
Touhoku	1951–1968	-1.51*	1968–1980	-3.30*	1980–1994	-4.22*	1994–2002	-5.84*		
Tyuubu	1951–1976	-1.45*	1967–1976	-2.73*	1976–1991	-4.60*	1991–1994	-2.78*	1994–2002	-5.95*
Tyuugoku	1951–1965	-1.53*	1965–1977	-3.00*	1977–1989	-5.00*	1989–1994	-3.13*	1994–2002	-6.41*
Causes of death										
Cerebrovascular	1955–1972	-1.07*	1972–1991	-4.79*	1991–1994	-1.75	1994–1997	-10.43*	1997–2002	-7.47*
Heart disease	1995–1966	-0.96*	1966–1969	-5.51*	1969–1977	-2.48*	1977–1989	-4.70*	1989–2002	-2.54*
Cancer	1955–1970	-4.53*	1970–1982	-9.55*	1982–1990	-7.78*	1990–1994	3.54*	1994–2002	-2.04*
Age										
65–74	1980–1989	-8.77*	1989–1997	-3.15*	1997–2002	-0.19				
75–84	1980–1990	-7.45*	1990–1994	-3.50*	1994–1997	-9.04*	1997–2002	-4.31*		
85 and over EU	1980–1989	-5.19*	1989–1994	-3.71*	1994–2000	-9.80*	2000–2002	-7.29*		

^1^APC is the annual percent change.

The APC is statistically significantly different from 0 (two-side p < 0.05). * p < 0.05

NOTE: Joinpoint, APC, and significant of APC are estimated using joinpoint analysis (JPA). The maximum of four joinpoints and five line segments was allowed for each long-term trend. In addition, segments had to contain at least two observed data points, and no segment could begin or end closer than two data points from the beginning or end of the data series.

Figure 1(B) shows the trend of home deaths by region. A significant decrease in the percentage of patients dying at home was observed in all 8 regions. The Hokkaido region had the lowest home death rate compared with other areas. The Kyuusyu region also kept a lower home death rate.

Home deaths by cause of death are shown in Figure 1(C). The probability of death at home among the patients dying from cerebrovascular and heart disease decreased significantly in recent 2 decades. The model for cerebrovascular indicated 4 joinpoints, and a more steep decline since 1994. The model for cancer was marked 4 joinpoints in 1970, 1982, 1990 and 1994. The home death rate first fell (APC = -4.53. -9.55, -7.78), and an increased trend was observed from the beginning of 1990's. (1990–1994, APC = 3.54).

The percentage of home deaths by age in the elderly is presented in Figure 1(D). The proportion of home deaths decreased over time in each age group of the elderly who died of the three leading causes. (cerebrovascular disease, heart disease and cancer).

Table 3 shows Odds of dying at home compared to odds of dying in other settings and 95% confidence intervals from multivariable logistic regression analysis. A significant decrease in the percentage of patients dying at home in the study period was observed. Odds ratios of home deaths were lowest (compared with the reference category of those dying during the period from 1980–1984) for those dying during the 2000–2002 period (OR = 0.263, 95% CI, 0.262–0.265). Decedents with heart disease and cerebrovascular had higher proportions of home deaths compared with those dying of cancer (for heart disease OR = 4.309, 95% CI, 4.291–4.327; for cerebrovascular OR = 3.616, 95% CI, 3.600–3.631). Age shows an increasing trend of probability of home death with increasing age at death. When gender, death year and course of death were adjusted, the home death rate in the group aged over 85 years was three times higher than that in the under 65 group (OR = 3.54, 95% CI, 3.52–3.55). The result of logistic regression analysis indicated that men were more likely to die at home than women (OR = 1.08, 95% CI, 1.074–1.08).

**Table 3:** Adjusted Odds of dying at home compared to odds of dying in other settings and 95% confidence intervals for patient characteristics from multivariable logistic regression analysis for subjects dying of cancer, heart disease, and cerebrovascular disease from 1980–2002.

**Variables**	b*	**Wald Chi-square statistics**	**P**	**Odds Ratios**	**95% Confidence Interval**
Intercept	-2.263	562139.321	< 0.0001		
**Year of death**					
1980–1984				1	
1985–1989	-0.488	46346.315	< 0.0001	0.614	0.611–0.617
1990–1994	-0.798	119217.658	< 0.0001	0.450	0.448–0.452
1995–1999	-1.095	209436.362	< 0.0001	0.334	0.333–0.336
2000–2002	-1.334	209958.211	< 0.0001	0.263	0.262–0.265
**Age**					
Under 65				1	
65–74	0.263	8926.060	< 0.0001	1.300	1.293–1.307
75–84	0.777	97556.695	< 0.0001	2.174	2.164–2.185
85 or over	1.262	225213.075	< 0.0001	3.534	3.515–3.552
**Gender**					
Female				1	
Male	0.075	2204.343	< 0.0001	1.078	1.074–1.081
**Cause of death**					
Cancer				1	
Cerebrovascular	1.285	351250.089	< 0.0001	3.616	3.600–3.631
Heart disease	1.461	476921.234	< 0.0001	4.309	4.291–4.327
					
-2log likelihood	10113580				
Chi-square model (df = 10)	1467394.7				
P	< 0.0001				
Overall rate of correct classification	74.0%				

* Unstandardized logistic regression coefficients

The results of correlation analysis and multiple regression analysis are presented in Tables 4 and 5.

**Table 4:** Spearman correlation coefficients between variables and home deaths rate among 47 prefectures.

**Variables**	**Spearman coefficient**	**p-value**
The number of general hospitals	-0.580*	< 0.001
The number of beds in hospital	-0.562*	< 0.001
Ratio of daily occupied beds in general hospital	-0.457*	0.001
The number of families in which the elderly living alone	-0.421*	0.003
Dwelling rooms (per dwelling)	0.440*	0.002
The number of health service facilities for the elderly	-0.495*	< 0.001

*P < 0.01

**Table 5:** Result of stepwise multiple regression analysis Stepwise multiple regression analysis using home death rate by prefectures in 2002 as dependent variable, and the number of general hospitals, the number of beds in hospital, ratio of daily occupied beds in general hospital, the number of families in which the elderly living alone, dwelling rooms (per dwelling) and the number of health service facilities for the elderly as independent variables

Independent variables	B*	β**	t	P
Step 1				
The number of beds in hospital	0.004	-0.598	-5.003	< 0.001
R square	0.357	Adjusted R square		0.343
R square change	0.357	P < 0.001		
Step 2				
The number of beds in hospital	0.004	-0.626	-6.218	< 0.001
Dwelling rooms (per dwelling)	1.444	0.446	4.433	< 0.001
R square	0.556	Adjusted R square		0.536
R square change	0.198	P < 0.001		
Step 3				
The number of beds in hospital	0.005	-0.883	-6.602	< 0.001
Dwelling rooms (per dwelling)	2.383	0.736	5.161	< 0.001
The number of families in which the elderly living alone	0.184	0.543	2.706	0.10
R square	0.620	Adjusted R square		0.594
R square change	0.065	P = 0.010		
Step 4				
The number of beds in hospital	0.005	-0.763	-5.595	< 0.001
Dwelling rooms (per dwelling)	2.502	0.773	5.676	< 0.001
The number of families in which the elderly living alone	0.204	0.505	3.145	0.003
Ratio of daily occupied beds in general hospital	-0.192	-0.263	-2.402	0.021
R square	0.666	Adjusted R square		0.635
R square change	0.046	P = 0.021		

*Unstandardized coefficients **Standardized coefficients

Table 4 shows the Spearman's correlation coefficients between variables and home death rate. All the variables showed a statistically significant correlation with home death rate.

Results of multiple regression analysis of the variables that affected home deaths are given in Table 5. Step 1, which included only the number of beds in hospital, accounted for 34.3% of the variance (Adjusted R^2 ^= 0.343). The inclusion of the number dwelling rooms (per dwelling), the number of families in which the elderly living alone and ratio of daily occupied beds in general hospital into step 2, step 3 and step 4 respectively resulted in an additional 19.8%, 6.5% and 4.6% of the variance being explained (R square change in step 2, step 3 and step 4 was 0.198, 0.065 and 0.046). In the final step the adjusted R square statistic was 0.635, indicating that 63.5% of the variance was accounted for in this regression model.

## Discussion

This study is based on vital statistic data in Japan. It is the best data currently available to study the differences and trends concerning the place of death the nations. Trend analysis indicated a significant decline during the 20th century. The reduction in home deaths may be related to multiple changes. The 1960s to the late 1980s was a period of rapid growth in health care services in Japan, during which the numbers of hospital, doctors, nurses and hospital beds increased rapidly. Changes in the treatments available to manage difficult symptoms, along with changes in social and clinical circumstances occurred during this period. Results from previous research have illustrated that regional differences in utilization and availability of health care services, and scope of coverage might influence the place of death [1,12,13]. Pritchard [12] found that almost all of the variance in place of death was explained by the hospital-day rate (hospital days per 1,000 beneficiaries), and by the number of acute care beds (per 1,000 population). In regions with more hospital beds and a higher rate of bed use, the in-hospital death rate was significantly higher. The area of residence was the most powerful predictor of where patients died, and risk of hospital death was significantly associated with several characteristics of the local heath system.

Hunt's study [27] in Australia also indicated that most of the institutionalization of death up to 1970 appeared to be due to increasing proportions of death in the growing number of public hospital beds. Thorne and colleagues [28] indicated that the presence of community hospital beds was associated with a significant reduction in home deaths. In our study, we also demonstrated this impact on home deaths. Decreasing home deaths was related to increasing number of beds in hospital and the utilization of the hospital.

Joinpoint regression provided us an objective method to evaluate the changes in the trend of home deaths. In this paper we used this method to demonstrate a significant decrease in home death and a significant increase in hospital death. Here, we only explain the four joinpoints occurred in home death trend for all death. As mentioned above, from 1960's there was a rapid increase in hospital, doctor and bed in Japan. It would contribute to the first joinpoint occurred in the middle of 1960's. The second join-point in the beginning of 1970's might be related with the setting of a payment system for old age medical care cost in 1973. By this system the government paid for the medical care cost which should be paid for by the elderly themselves. This policy increased the usage of medical health resources among the elderly and resulted more patients died at hospital. The number of hospital and bed turned to be saturated at the end of 1980's. It might partly explain why the speed of decrease in home death rate became slowly since late 1980's. In 1994, home for the elderly was added as an independent category of site of death in the Vital Statistics, and from 1994 to 2002 the proportion of death at home for the elderly increased from 1.5% to 1.9%. Because before 1994, part of death at home for the elderly was classified as death at home, the changed trend which has appeared since 1994 seems reasonable.

Changes in Japanese society since the 1950s have been mainly a large increase in the number of families where elderly people are living alone. The ability to give care at home has been considerably reduced. Some studies indicate that the availability of care givers or strong family support is important in facilitating in-home deaths [29,30]. Our findings also demonstrated that the proportion of the families with the elderly living alone bore a negative correlation with home death rates.

The first modern palliative care service available in Japan was launched in 1971. The Japanese Government's Ministry of Health, Labor and Welfare granted approval for coverage of inpatient hospice/palliative care services by the National Medical Insurance in 1991, and the number of inpatient hospices and palliative care units increased from 5 in 1991 to 104 in 2004. These hospital-based special palliative care units provide treatment oriented toward the resolution of symptoms, along with a home-like living environment. Some of them even offer a traditional style tatami-floored room (traditional Japanese reed mats used as floor covering) to satisfy the patient's wishes to die on tatami floors. These efforts might partly account for the increasing hospital death rates, especially for cancer patients, in Japan in recent decades. We were unable to obtain information about how many patients who accessed hospital hospice units before death eventually died at home, and it is difficult to discriminate the real impact of the hospice on where people die. Home hospice care service in Japan is not a mainstream style of palliative care, and lags behind similar care in Western countries[31,32] The impact of these facilities on home deaths needs to be clarified.

For home deaths by geographic difference, regretfully, we could not put geographic variables (urban/rural and region) into logistic regression model, because the data used in logistic regression analysis was category table data, and the geographic variables (urban/rural and region) were not included in the category tables. Although the absence of statistical test for geographic difference is a shortcoming, our study still showed that the Hokkaido, Kyuusyu region, held the lower home death rate. Geographic differences might be explained by the different distribution of medical services resources. The two regions mentioned above were areas with more hospitals and hospital beds compared with other regions. This may increase the odds that terminal patients are placed in a medical facility other than kept at home for end-of-life care or death. Further studies using death certification data to demonstrate the geographic difference in home death and the study to testify these assumptions mentioned above are necessary.

We also found a higher probability of home deaths with increasing age. This result is consistent with other reports performed in Australia and USA. Hunt [27] found that in Australia, younger patients were more likely to die in a public hospital, and explained that it was possibly because of a tendency to treat younger patients more actively. By examining the longitudinal trend of home deaths, we found that variation in home deaths by age decreased over time. This trend may result because new treatments have become available and these improvements in medicine have allowed elderly, terminally ill patients to access treatment more activity over time.

In our study, males had an increased rate of home death. Similar results were found in other studies [27,29,33-36]. In contrast, Costantini and colleagues [37] found that the proportion of home deaths was significantly higher among females than among males. A number of explanations are possible for these gender differences in place of death. First, there may be true differences between male and female in their preferences for place of death. Regrettably, in this study no information is available about preferences at the end of life. Second, there may be similar preference, but there are differences in the possession of other resources necessary to fulfill preferences. Third, men may be able to obtain home care more easily than women. Published results have indicated that the primary informal care-givers were predominantly female, and although women provide the majority of informal end-of-life care, when women themselves need care, they receive less assistance from family members than men do [38,39]. In our study, this gender difference should be examined carefully, because the large sample size may overpower the statistical significance easily.

There are conflicting reports on whether differences in the site of death is correlated with the cause of death [13,27,38] Hunt et al. [27] found that there was no difference in the site of death for patients dying of cancer and non-cancer. However, Fried [13] found that for elderly patients in community-based long-term care programs, those with cancer were less likely to die in the hospital. Results from SUPPORT showed that people with colon cancer were less likely to die in the hospital than those who suffered from other illnesses [12]. The findings from our study suggest that there are significant differences among different causes of death, and cerebrovascular and heart disease were more likely to occur at home as compared with death from cancer. This pattern did not change over the period examined in this study. The burden of sustained home care and the tremendous financial burden for patients with cancer may cause them to choose to die at a place other than the home. Moreover, it is very difficult to control severe pain at home. The family of the cancer patient with pain may send the patient to the hospital regardless of his or her desire. An increased rate of home deaths will be seen when cancer patients can access home hospice care easily.

There are some limitations to the results presented here. First, because of the large sample size of the study, statistical analyses are overpowered. Examining the magnitude of the trend is more important than focusing on statistical significance. Second, there are some limitations of ecological studies, such as weak indicators or a lack of indicators of potential confounders. Third, death data used in this study come from published document. It is a kind of summary table data, not death certification. Because there are only few variables in the category table, it is not possible to include all the factors in a model simultaneously. However, since there is little research focused on factors of social and health/welfare services related to home deaths in Japan, we feel the information presented here is pertinent.

## Conclusion

The pattern of the place of death has not only been determined by social and demographic characteristics of the decedent, but also associated with the medical service in the community. Concerning the great disparity between the preference for home deaths and the reality that most deaths occur in hospitals in Japan, we believe that our findings still have important implications for the policy of health care services. These implications include: first, because most of patients die in hospital setting, the establishment of specialized palliative consulting teams in hospitals is of important. Hospital palliative consulting teams should work with hospice and community based programs to provide integrated care and to promote better community service. Second, because home death has a close relationship with the ability to providing home care before death, home care services and specialized palliative care-services should provide full responsibility for the patients' treatment and care; especially for the elderly living alone, and these factors might enable more time at home and more home death.

## Competing interests

The author(s) declare that they have no competing interests.

## Authors' contributions

LY participated in the design of the study, performed the statistical analysis, and drafted the manuscript. NS was involved in collecting data and helped to draft the manuscript. EM participated in its design and was also involved in revising the manuscript. All authors read and approved the final manuscript.

## Pre-publication history

The pre-publication history for this paper can be accessed here:


